# Use of antibiotics by adults: a population-based cross-sectional study

**DOI:** 10.1590/1516-3180.2018.0168060818

**Published:** 2018-10-22

**Authors:** Jéssica Quintão Pereira, Marcus Tolentino Silva, Taís Freire Galvão

**Affiliations:** I BPharm. Pharmacist and Assistant Scientist at Johnson & Johnson Brasil, São José dos Campos (SP), Brazil.; II MSc, PhD. Pharmacist and Professor, School of Medicine, Universidade Federal do Amazonas (UFAM), Manaus (AM), Brazil, and Postgraduate Program on Pharmaceutical Sciences, Universidade de Sorocaba (UNISO), Sorocaba (SP), Brazil.; III MSc, PhD. Pharmacist and Professor, School of Pharmaceutical Sciences, Universidade Estadual de Campinas (UNICAMP), Campinas (SP), Brazil.

**Keywords:** Anti-bacterial agents, Drug utilization, Cross-sectional studies, Self-medication

## Abstract

**BACKGROUND:**

The consumption of antibiotics has been widely discussed, mainly because of antibacterial resistance, which has become a worldwide concern. In Brazil, sale of antibiotics is currently ruled by Agência Nacional de Vigilância Sanitária (ANVISA) regulation RDC 20/2011, which restricts sales to those made under medical prescription. The aims of this study were to evaluate antibiotic use and associated factors among adults in the Metropolitan Region of Manaus, Amazonas, Brazil, and to assess the proportion of self-medication from this use.

**DESIGN AND SETTING:**

Population-based cross-sectional study conducted in the Metropolitan Region of Manaus between May and August 2015.

**METHODS:**

Adults aged ≥ 18 years were selected through probabilistic sampling in three stages. Trained interviewers collected data from the participants in their homes. Antibiotic consumption over the last 15 days was reported. Bivariate analysis was used to calculate the prevalence ratio (PR) of antibiotic usage, with 95% confidence interval (95% CI). A multivariate model adjusted according to significant variables at P ≤ 0.20 using Poisson regression with robust variance was constructed.

**RESULTS:**

The prevalence of antibiotic use was 3.4% (95% CI 2.8-4.0%). Adjusted analysis showed that consumption was higher among women than among men (PR 1.58; 95% CI 1.11-2.24) and among people with fair health status than among those with good health (PR 1.52; 95% CI 1.08-2.15). The prevalence of self-medication was 19.0%; amoxicillin was the most self-medicated antibiotic (10/26).

**CONCLUSION:**

Antibiotic use was associated with women and individuals with fair health status. One fifth of the antibiotics were consumed through self-medication, contrary to the current Brazilian legislation.

## INTRODUCTION

Antimicrobials are medicines that kill or prevent the growth of pathological microorganisms in the body. Among antimicrobial agents, antibiotics are among the most common drugs used worldwide.^1^ Antibiotic therapy is intended for treatment of potential or proven bacterial infections. Administration of antibiotics consists of a set of actions that aims to optimize their use, through choosing the most appropriate medicine for a particular treatment and its dosage, route and time of administration, as well as minimizing undesirable events, such as toxicity and bacterial resistance.^2^^,^^3^ This makes it very important to analyze the profile of the population exposed to these agents, since their action may be affected by gender, age and other factors.

Use of antimicrobials is associated with the threat of antimicrobial resistance. Different sources and transmission routes influence antimicrobial resistance, including healthcare, agriculture and the natural environment.^4^ It has been estimated conservatively that 700,000 deaths per year occur consequently to antimicrobial resistance and that by 2050, there might be 10 million deaths per year. Consequently, there will be economic losses of 100 trillion dollars due to resistant infections worldwide, if the current scenario does not change.^5^ In Brazil, sale of antibiotics has been regulated since 2010, initially through resolution (Resolução de Diretoria Colegiada, RDC) number 44/2010 of the Brazilian Health Regulatory Agency (Agência Nacional de Vigilância Sanitária, ANVISA), and subsequently through ANVISA resolution RDC 20/2011, which remains in force. RDC 20/2011 specifies that the sale is only authorized upon presentation of two copies of a medical prescription valid for 10 days, of which one will be kept by the pharmacist.^6^

Despite the requirement for a medical prescription, self-medication is often reported. A population-based cross-sectional study conducted in the state of Goiás in 2008 observed that 7% of the interviewees used antibiotics in the month prior to the interview, and that 9% of this usage occurred without prescription.^7^ In addition, use for a longer or shorter time than indicated was common, thus potentially contributing towards bacterial resistance.

No population-based studies evaluating the consumption of antibiotics after their use became regulated in Brazil are available. Assessment of the consumption of these products by the population and the proportion of nonprescription use is important for measuring the extent of exposure and the effect of the legislation on people’s health.

## OBJECTIVE

The purpose of this study was to evaluate the prevalence of antibiotic consumption and associated factors among adults in a metropolitan region in northern Brazil, and to estimate the rate of self-medication.

## METHODS

### Design

This was a population-based cross-sectional study conducted between May and August 2015 among adults living in the Metropolitan Region of Manaus, which is located in the northern region of Brazil. This study formed part of a larger research project that aimed to evaluate the use of healthcare inputs and services.^8^

### Setting

The Metropolitan Region of Manaus is composed of the capital of the state of Amazonas (Manaus), and seven other municipalities: Careiro da Várzea, Iranduba, Manacapuru, Itacoatiara, Novo Airão, Presidente Figueiredo and Rio Preto da Eva. In 2010, this region had over 2.1 million inhabitants, i.e. more than 60% of the population of Amazonas. In 2010, this region occupied 19^th^ position in the Brazilian ranking of the Municipal Human Development Index (Índice de Desenvolvimento Humano Municipal, IDHM), out of a total of 20 metropolitan regions in Brazil, among which São Paulo was in first position.^9^

### Participants and sample size

Adults of at least 18 years of age who were living in the region were eligible for inclusion. We calculated the sample size as 4,000 adults to be interviewed, through considering the following factors: 2,106,322 adults living in the region, 50% healthcare service utilization rate, 95% confidence level, 2% accuracy and design effect of 1.5, with an addition of 10% to compensate for losses.^8^

The participants were selected by means of probabilistic sampling in three stages. From 2,647 urban census tracts, 400 primary and 20 secondary census tracts were selected through probabilistic sampling. The households were selected by means of systematic sampling: the first household was drawn and, from this, every 20^th^ house in the same street was systematically visited. In cases of refusal or unavailability, the house immediately to the right was visited, and the same process was repeated to the left, if necessary. The third step was to select the participant to be interviewed: all adults present in the house were registered and one was drawn in accordance with pre-defined age and sex quotas, so as to reach population representativeness.

### Variables, data sources and measurement

The data were collected in the participants’ homes by 14 trained interviewers using electronic devices, after each participant had agreed to participate in the survey by signing an informed consent form.

The primary outcome was the use of antibiotics over the last 15 days. The independent variables included: sex (female or male); age (in years); social class according to the Brazilian Economic Classification Criteria (A, B, C or D/E), which is based on the number of household appliances, number of domestic employees, level of education of the head of the family and access to urban services;^10^ self-assessment of health status (good, fair or poor); and use of healthcare services over the last 15 days (yes or no).

The use of medicines over the previous 15 days was measured using the question *“Over the past 15 days (or two weeks) did you take any medicine?”*. In cases of a positive response, the name of the medicine, the disease or health problem, the length of time for which the medicine was used, the person responsible for indication and the form of acquisition of the medicine were recorded as reported by the interviewee. After the data had been tabulated, the names of the medicines were surveyed in accordance with the Brazilian list of common names and were then classified using the Anatomical Therapeutic Chemical (ATC) Classification System of the World Health Organization.^11^ Medicines that remained unidentified, either due to an indecipherable name or due to not being listed in the ATC were classified as “non-codifiable”.

The indication and source of acquisition of the antibiotic was measured by the question: *“Who prescribed it?”* (physician, sales clerk, relatives/neighbors, interviewee upon his/her own account, pharmacist or other) and *“What was the form of acquisition of the medicine?”* (health insurance, *Farmácia Popular* (public co-payment program), public healthcare system, drug store or other)*.* A situation of self-medication was recorded when the person responsible for antibiotic indication was not a physician.

After the data had been tabulated, interviews in which use of antibiotics was reported were identified in the database in accordance with the list of medicines included in ANVISA regulation RDC 20/2011. These products corresponded to the antimicrobials that are included in group J of the ATC, i.e. antiinfectives for systemic use; specifically, in the groups J01 (J01A tetracyclines, J01B amphenicols, J01C beta-lactam antibacterials, penicillins, J01D other beta-lactam antibacterials, J01E sulfonamides and trimethoprim, J01F macrolides, lincosamides and streptogramins, J01G aminoglycoside antibacterials, J01M quinolone antibacterials, J01X other antibacterials) and J04 (antimycobacterials: J04A drugs for treatment of tuberculosis and J04B drugs for treatment of leprosy).^11^

### Statistical methods

Initially, descriptive statistics were obtained from the variables of the study, and the absolute and relative frequencies of each variable and the frequency of antibiotic use per variable were calculated. Poisson regression with robust variance was performed to calculate the prevalence ratios (PR) of antibiotic use, with the 95% confidence interval (95% CI). Firstly, we performed bivariate analysis: the variables that were significant at the level P ≤ 0.20 were included in the multivariate model to calculate adjusted PRs. Associations were considered significant if P < 0.05. The Wald test was used to calculate the P-values of the variables. The analyses were performed using the Stata 14.2 software, taking into account the complex sampling design (svy command).

### Ethics

The Research Ethics Committee of the Federal University of Amazonas (Universidade Federal do Amazonas, UFAM) approved the project, through report no. 974,428 of March 3, 2015 (certificate of presentation for ethics assessment on the Brazil Platform [CAAE] 42203615.4.0000.5020).

## RESULTS

The survey included 4,001 adults, of whom 136 reported consuming antibiotics, at the rate of one antibiotic per person. Thus, the prevalence of antibiotic consumption over the 15 days prior to the interview was 3.4% (95% CI: 2.8-4.0%).

The study population showed slight predominance of women (52.8%) and individuals between 25 and 34 years of age (28.8%), who were in economic class C (57.1%), who had completed high school (47.5%), who were in good health (66.1%) and who had not used any healthcare services over the 15 days prior to the interview (79.0%).

The frequency of use was higher among women (4.2%) than among men (2.5%), and among adults aged 25 to 34 (3.8%) and 35 to 44 (3.7%), while use was less frequent among the elderly (2.8%). Consumption was higher among the poorest population (D/E, 4.1%), and among individuals who reported having fair health (4.6%), as shown in Table 1.

**Table 1.** Main characteristics of the population and frequency of antibiotic consumption, adjusted for the complex sampling design. Metropolitan Region of Manaus, 2015 (n = 4,001)

Characteristics	Population		Antibiotic consumption	
	n	%	n	%
Sex				
Male	1,888	47.2	47	2.5
Female	2,133	52.8	89	4.2
Age (years)				
18-24	838	20.9	28	3.4
25-34	1,152	28.8	44	3.8
35-44	843	21.1	31	3.7
45-59	772	19.3	22	2.9
60 and over	396	9.9	11	2.8
Social class				
A/B	269	15.7	15	2.4
C	2,285	57.1	77	3.4
D/E	1,087	27.1	44	4.1
Education				
Higher education or above	158	4.0	7	2.5
High school	1,903	47.5	74	3.9
Elementary school	649	16.2	14	2.6
Less than elementary school	1,291	32.3	41	3.2
Health status				
Good	2,646	66.1	75	2.8
Fair	1,108	27.7	15	4.6
Poor	247	6.2	10	4.0
Healthcare service use over the last 15 days				
Did not use	3,163	79.0	104	3.3
Used	838	21.0	32	3.8

In the bivariate analysis (Table 2), higher consumption of antibiotics was observed among women (PR 1.70; 95% CI 1.20-2.40), people with fair health (PR 1.63; 95% CI 1.15-2.32) and people in social class D/E (PR 1.71; 95% CI 0.96-3.05).

**Table 2.** Unadjusted and adjusted prevalence ratios (PRs) and 95% confidence interval (CI) of antibiotic use according to study variables. Metropolitan Region of Manaus, 2015 (n = 4,001)

Characteristics	PR (95% CI)	P-value	Adjusted PR (95% CI)	P-value
Sex		<0.001		0.011
Male	1.00		1.00	
Female	1.70 (1.20-2.40)		1.58 (1.11-2.24)	
Age (years)		0.731		
18-24	1.00			
25-34	1.15 (0.72-1.83)			
35-44	1.09 (0.66-1.81)			
45-59	0.85 (0.49-1.47)			
60 and over	0.83 (0.42-1.64)			
Social class		0.181		0.393
A/B	1.00		1.00	
C	1.41 (0.82-2.44)		1.32 (0.77-2.28)	
D/E	1.71 (0.96-3.05)		1.50 (0.84-2.67)	
Education		0.379		
Higher education or above	1.00			
High school	1.55 (0.57-4.18)			
Elementary school	1.04 (0.36-3.06)			
Less than elementary school	1.27 (0.46-3.51)			
Health status		0.020		0.059
Good	1.00		1.00	
Fair	1.63 (1.15-2.32)		1.52 (1.08-2.15)	
Poor	1.42 (0.75-2.72)		1.28 (0.66-2.47)	
Healthcare service use over the last 15 days		0.465		
Did not use	1.00			
Used	1.16 (0.78-1.71)			

In the multivariate analysis, which was adjusted according to sex, health status and social class, use of antibiotics were higher among women than among men (PR 1.58; 95% CI 1.11-2.24) and among people with fair health status in comparison with good health status (PR 0.52; 95% CI 1.08-2.15). No association was found between antibiotic consumption and age, social class, education level or use of healthcare services over the previous 15 days.

Among the 136 people who used an antibiotic, a medical prescription was the main form of indication (81.0%; Table 3), while 19% used an antibiotic through self-medication, without a medical prescription. Use upon the interviewee’s own account totaled 13.2%, while use upon suggestion from relatives and neighbors comprised 5.1% and from a pharmacist, 0.7%. Purchases of antibiotics without a prescription were self-reported. No further investigation was made about how the individuals could purchase the medication without a physician prescription. Cephalexin (39.7%) and amoxicillin (29.4%) were the drugs most used, followed by benzathine benzylpenicillin (4.4%), ciprofloxacin (3.7%), sulfadiazine (3.7%), tetracycline (3.7%), azithromycin (2.9%) and levofloxacin (2.9%). There were 10 cases of self-medication with amoxicillin, six with cephalexin and four with tetracycline, which are the antibiotics most commonly consumed without medical prescription. There were also three cases with sulfadiazine, and one each with benzylpenicillin, azithromycin and levofloxacin (Table 4).

**Table 3.** Person responsible for indication of the antibiotics consumed over the 15 days prior to the interview. Metropolitan Region of Manaus, 2015 (n = 136)

Person responsible for indication	n	%
Physician	110	81.0
Interviewee (upon own account)	18	13.2
Relatives/neighbors	7	5.1
Pharmacist	1	0.7
Total	136	100

**Table 4.** Total frequencies of antibiotic consumption and numbers of cases of self-medication with each drug over the last 15 days prior to the interview. Metropolitan Region of Manaus, 2015 (n = 136)

Medicine	ATC code	N	%	N self-medication
Cefalexin	J01DB01	54	39.0	6
Amoxicillin	J01CA04	40	29.4	10
Benzylpenicillin benzathine	J01CE01	6	4.4	1
Ciprofloxacin	J01MA02	5	3.7	-
Sulfadiazine	J01EC02	5	3.7	3
Tetracycline	J01AQA07	5	3.7	4
Azithromycin	J01FA10	4	2.9	1
Levofloxacin	J01MA12	4	2.9	1
Sulfamethoxazole + trimethoprim	J01EE01	3	2.2	-
Ampicillin	J01CA01	2	1.5	-
Norfloxacin	J01MA06	2	1.5	-
Erythromycin	J01FA01	1	0.7	-
Ethambutol	J04AK02	1	0.7	-
Methenamine	J01XX05	1	0.7	
Nitrofurantoin	J01XE01	1	0.7	-
Pyrazinamide	J04AK01	1	0.7	-
Rifampicin	J04AB02	1	0.7	

ATC = anatomical therapeutic chemical classification system.

## DISCUSSION

Out of every 100 adults in the Metropolitan Region of Manaus, three to four used some kind of antibiotic over the 15 days preceding the interview. Consumption was higher among women and among people with fair health. Approximately one fifth of the antibiotics consumed were through self-medication, thus highlighting the existence of weaknesses in the regulatory control over these products.

All the data of the present study, including in relation to self-medication, relied on self-reports from the interviewees. The number of people using antibiotics and the true number of medicines recorded may have been higher than what was observed, since the participants could have forgotten to report their use of a medication. In addition, the recording of product names by the research team may not have been adequate in some cases, since medicine packaging or medical prescriptions were not personally verified during the interviews.

Another limiting factor was the fact that the pediatric population, which is an age group that presents high consumption of antibiotics, was not included in this study. According to an analysis on a French database of healthcare service use between 2010 and 2011, about 80% of the children aged 0 to 24 months who presented recurrent coryza consumed some type of antibiotic over a six-month period.^12^ Another study conducted in public schools in Chicago in 2004 noted that 40% of the children and adolescents aged 4 to 18 were using antibiotics to treat asthma or recurrent coryza.^13^ A household survey on the use of antibiotics among children, teenagers and adults in the United States found that out of 71,444 subjects, approximately 5% had had an antibiotic prescribed between 1999 and 2012.^14^

Seasonality affects the consumption of antibiotics: the highest frequency of prescription is usually reported during the fall (autumn) and winter,^15^^,^^16^ seasons that occur between March and September in most of Brazil. The state of Amazonas presents an equatorial climate, characterized by average temperatures of 26 to 28 °C throughout the year and two seasons: rainy (December to May) and dry (June to November). Particularly in Manaus, there is a predominance of high temperatures during the rainy or “winter” season (December to May), with maximum temperatures of 31 to 33 °C.^17^

Higher consumption of antibiotics among women has also been observed in other studies.^18^^,^^19^^,^^20^ The habit of taking care of personal health is more common among women, which increases the rate of diagnosis and treatment of diseases in this sex. Furthermore, female anatomical and physiological characteristics favor susceptibility to certain infections, such as urinary tract infections.^21^ An international population-based study conducted on approximately 29 million adults in Denmark, Netherlands, Italy, United Kingdom and Germany noted important changes in consumption in these countries over the space of a decade or more, varying internally according to age and sex.^22^ In another population-based study conducted between 1998 and 2009 on urban and rural populations in the Netherlands, use of antibiotics was higher in the rural region and among women in both regions.^23^

Consumption of antibiotics has been associated with self-assessment of health as “fair”. Individuals with this health status are probably not seriously ill, but their health may be somewhat weakened due to a common cold, fever, pain or common respiratory tract infections. A study conducted in Vellore, southern India, analyzed prescriptions and dispensations in small hospitals, clinics and pharmacies from 2003 to 2005. It found that 41.0% of the drugs were antibiotics, and that their main uses were in cases of upper and lower respiratory tract infections, fever and diarrhea.^24^

In the current study, which was conducted five years after ANVISA introduced regulation of antibiotic sales, approximately one fifth of the antibiotics were used for self-medication. A Brazilian study conducted in 1998 involving 6,000 households in all regions of the country found similar self-medication rates.^25^ Analysis on antibiotic sales across Brazil between 2008 and 2012 showed that the change in sales policy regulation in 2010 had little impact in the north of the country, comprising a reduction in sales of around 7%. In the southern and southeastern regions of Brazil, which are more developed regions, these reductions were 13% and 16%, respectively.^26^ These reductions were more pronounced in the first year of the new regulation. In the following year, sales in the south and southeast continued to decrease, while in other regions sales increased again and reached almost the same level as before the legislation.^26^ An analysis on sales in the state of São Paulo over the same period (2008-2012) also found an immediate reduction in the consumption of oral antibiotics, which was increasing before enactment of the regulation, and found that a stable trend was reached in 2012.^27^ The legislation also had little impact on the quality and completion of medical prescriptions, which in over 90% of the cases were incomplete.^28^ No population-based studies conducted after implementation of regulation of antibiotic sales are available.

The antibiotics most consumed through self-medication in this analysis, i.e. amoxicillin, cephalexin and tetracycline, can be used to treat infections in the genitourinary tract,^29^ respiratory tract^30^^,^^31^ and skin.^32^ Such symptoms are recognized by the lay population, and this is potentially a factor favoring more frequent use of these medicines.

Self-medication is known to be one of the sources of bacterial resistance.^33^ Spreading of the disease and occurrences of adverse events are among the problems caused by self-medication, which negatively impacts not only the patient’s life, but also the healthcare system.^34^

## CONCLUSIONS

Use of antibiotics among adults in the Metropolitan Region of Manaus over the previous 15 days was higher among women and among people with fair health. Around one fifth of the antibiotics were reported to be used through self-medication, i.e. without a medical prescription, contrary to current Brazilian regulations. Enforcement of health regulatory inspections is needed to improve compliance with mandatory prescription of antibiotics and thus contribute towards a culture change that would promote rational use of antibiotics.
